# Foraminal widening indicating a spinal nerve tumor in the saber-tooth cat *Smilodon fatalis* from Rancho La Brea

**DOI:** 10.3389/fvets.2026.1857385

**Published:** 2026-07-27

**Authors:** Hugo Schmökel, Francesca Del Chicca, Regine Hagen Argudin Pina

**Affiliations:** 1 Evidensia Academy, Division of Veterinary Orthopaedics and Spine Surgery, Stockholm, Sweden; 2 Department of Paleontology, University of Zurich, Zurich, Switzerland; 3 Clinic for Diagnostic Imaging, Vetsuisse Faculty, University of Zurich, Zurich, Switzerland

**Keywords:** La Brea Tar Pits, nerve, pathology, Pleistocene, *Smilodon fatalis*, tumor, vertebra

## Abstract

Tracing the health of extinct populations is important. Documenting this in deep time is challenging, but skeletal markers of disease can be preserved in fossils. Notably, a high incidence of spinal malformations has been recorded among specimens found in the La Brea Tar Pits, possibly because of an accumulation of expressed deleterious alleles that caused disturbances in developmental processes. The exceptional preservation of hundreds of specimens of the La Brea saber-toothed cat *Smilodon fatalis* allows us to examine pathologies within and across populations, with dire wolves from the same site and extant large-bodied felids serving as a basis for comparison. We identified three vertebral specimens from *S. fatalis* with changes indicating a possible spinal nerve tumor (SNT). The prevalence-like estimate was 353 per 100,000 individuals. The observed prevalence of foraminal widening compatible with presumed SNT among the La Brea *S. fatalis* sample appears high relative to reported human incidence estimates of 0.22–0.38 per 100,000 individuals, although these values are not directly comparable because the fossil estimate reflects skeletal occurrence in a taphonomically biased assemblage rather than clinical incidence. The reason for this high incidence in *Smilodon fatalis* is unclear, but in humans, many SNTs are associated with genetic mutations. Due to the presumed inbreeding toward the end of the species’ existence, a similar mutation could have contributed to the development of the presumed SNTs in *S. fatalis.*

## Introduction

Spinal diseases such as tumors and intervertebral disc disease (IVDD) are well-documented in the domestic dog *Canis familiaris* and cat *Felis catus*. Some studies have examined the degree of involvement of wild carnivorans, in the wild as well as in captivity (1–8). Radiographic examination of live wild animals is costly, and, to the best of our knowledge, only a few institutions house wildlife postcranial skeletal collections large enough to permit reconstructing the prevalence of spinal disorders in a given population. Paradoxically, some extinct carnivorans are a rich source of information, being represented by hundreds of well-curated specimens from an exceptional fossil site: the Ice Age predators saber-toothed cat *Smilodon fatalis and the* dire wolf *Aenocyon dirus* (9–11). The La Brea tar seeps in Los Angeles, CA, USA, have preserved mostly disarticulated skeletons of these animals from the end of the Pleistocene. The tar seeps were a carnivore trap: a large herbivore that became mired in the asphalt inadvertently would attract large carnivores and scavengers, which would themselves become entrapped in great numbers. Many bone specimens were excavated over the last century from more than 100 tar deposits or “pits” that, together, present a picture of the several-thousand-year period leading up to the Late Pleistocene extinctions 12,000 years ago, when *Smilodon fatalis*, *Aenocyon dirus*, and other Pleistocene megafauna disappeared from North America (12–16).

The uncommonly large sample sizes enable documentation of the prevalence and distribution of articular, osseous, and vertebral diseases in the fossil species (9, 10, 17). The pathologies found in this Pleistocene predator can be compared to the spine pathologies of extant canids, large Felidae, and the domestic cat.

Tumors of the spine are generally rare and can be divided into primary spine tumors, secondary metastatic tumors, or multicentric neoplasia, which is more common in domestic cats than in dogs (18–22). In the vast majority of cases, foraminal enlargement is caused in humans by spinal nerve tumors (SNTs) including Schwannomas and neurofibromas of the nerve root passing through the affected foramen (19, 23, 24). In dogs, SNTs are rare, and they are often slow-growing benign Schwannomas and neurofibromas that cause a similar widening effect of the foramen as in humans. Malignant SNTs have been described in dogs too (19, 20). These tumors are very rare in domestic cats (21, 25). An enlarged nerve and an enlarged foramen are radiologically indicative of SNT in small animal radiology as they are in human medicine, usually due to a slow expansile rather than invasive pattern of growth.

This study aimed to describe three vertebral specimens with typical changes indicating a possible SNT in the saber-toothed cat *Smilodon fatalis.*

## Materials and methods

According to the La Brea Tar Pit Museum (LBTPM) database, the vertebrae of nearly 1,000 *S. fatalis* are preserved well enough in the collection to make it possible to examine them for trauma-related changes, malformations, secondary chronic degenerative changes, and changes caused by infection or tumors.

We examined pathological vertebrae and sacra found in pits 2, 3, 4, 13, 16, 60, 61/67, 77, and 91 and the pits ‘Academy’ and ‘General locality’. The analyzed specimens are housed at LBTPM in Los Angeles, CA, USA. Damaged and juvenile specimens, for which pathologies could not be accurately identified, were excluded from the analysis. A total of 849 *S. fatalis* sacra (the most common vertebral segment), 2,130 lumbar vertebrae, and 743 C7 vertebrae were examined using optical methods and computed tomography (CT) for this study. The minimal number of individuals (MNI = 849) used for the pathological occurrence calculations was based on the number of sacra, the most numerous single part of the spinal column in the La Brea *S. fatalis* collection. Pathologies were recorded and described, including malformations, bone proliferations along the vertebra, vertebral and facet joint fusions, and changes in the size of intervertebral foramina (10).

In total, 49 CT scans of *S. fatalis* spinal specimens with pathologies were performed at the Veterinary Teaching Hospital of the University of California, Davis, CA, USA, using a GE LightSpeed 16-slice CT Scanner providing 0.63 mm micro-Voxel™ resolution (GE HealthCare Technologies, Inc., Chicago, IL, USA). The images were reviewed by two board-certified veterinary radiologists (FDC and RHAP) using Horos v.3.3.6^1^ or OsiriX MD® DICOM viewing software (Pixmeo SARL, 266 Rue de Bernex, CH1233 Bernex, Switzerland). CT scans were reviewed in three orthogonal image planes after multiplanar reconstruction and in volume-rendered reconstructed images. Vertebral bodies were assessed for the presence of anomalies such as block vertebrae or hemivertebra formation, bony proliferation, or trauma such as fractures or fissures, and abnormally sized foramina.

## Results

In addition to a variety of spondyloarthropathies, 226 congenital transitional vertebral malformations were found; 32 bifid thoracic vertebrae and 48 block vertebrae were identified and confirmed using CT examinations (10). An additional lesion recorded was an enlarged intervertebral foramen in three specimens (Table 1). The three specimens were found in different pits and therefore represent three different individuals.

**Table 1:** *Smilodon fatalis* specimen with a widened vertebral foramen (ybp = years before present).

Specimen	Pit	Vertebra	Finding	Documentation
HC 12233	67; 12,000 ybp	L 6–7 left	Enlarged foramen	Photograph
HC 8253	77; 28,000 ybp	L3–4 left	Enlarged foramen, bone proliferations	CT, photograph
HC 12288	3; 13,000 ybp	L4–5 left	Enlarged foramen, bone proliferations	CT, photograph

**Figure 1:**
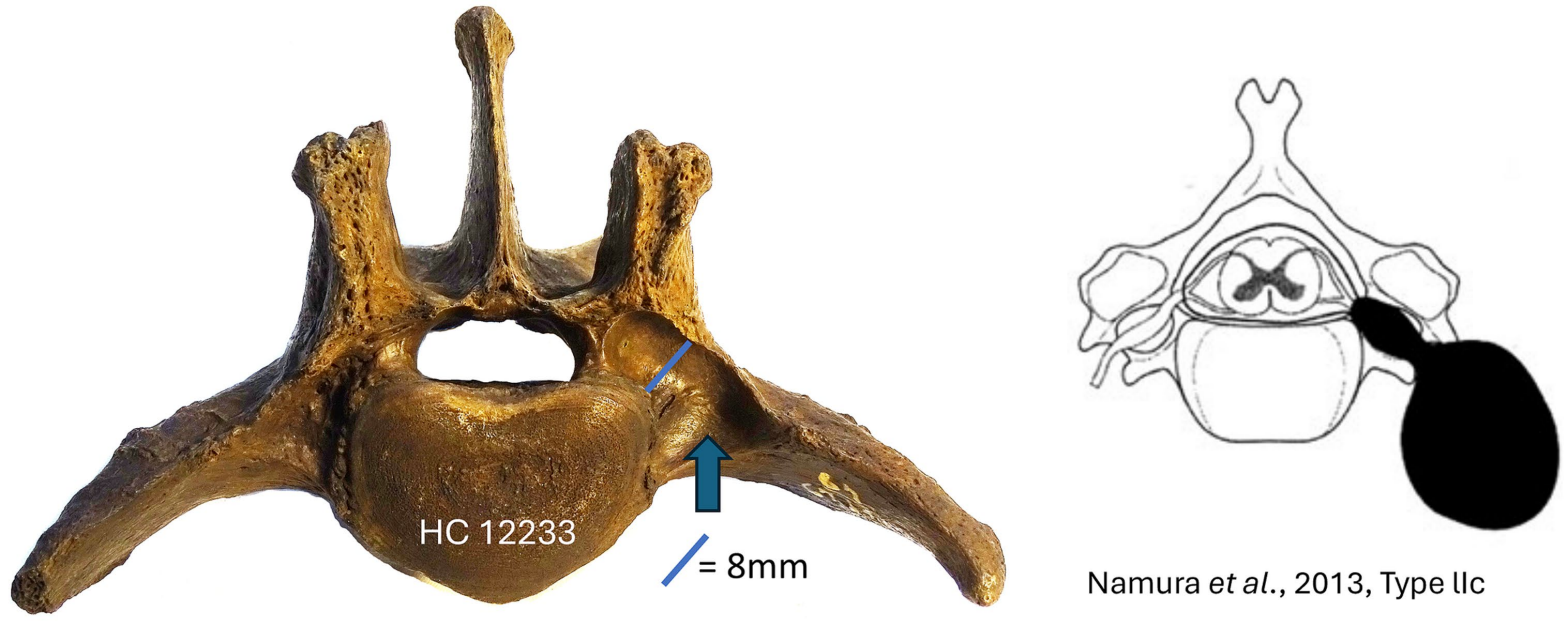
Photograph of the cranial L7 of HC 12233 showing a smoothly outlined left-sided foraminal widening and bone remodeling forming a ‘cast’-like indentation (arrow) of a presumed SNT (black space-occupying lesion in the drawing) at the level of the foramen.

In specimen HC 12233 (L7), the cranial part of the left intervertebral foramen is enlarged to twice the size of the right foramen, and distal to the foramen, a slight but clear imprint of a possibly nodular structure is visible (Figure 1). This may indicate the presence of a slowly growing SNT.

**Figure 2:**
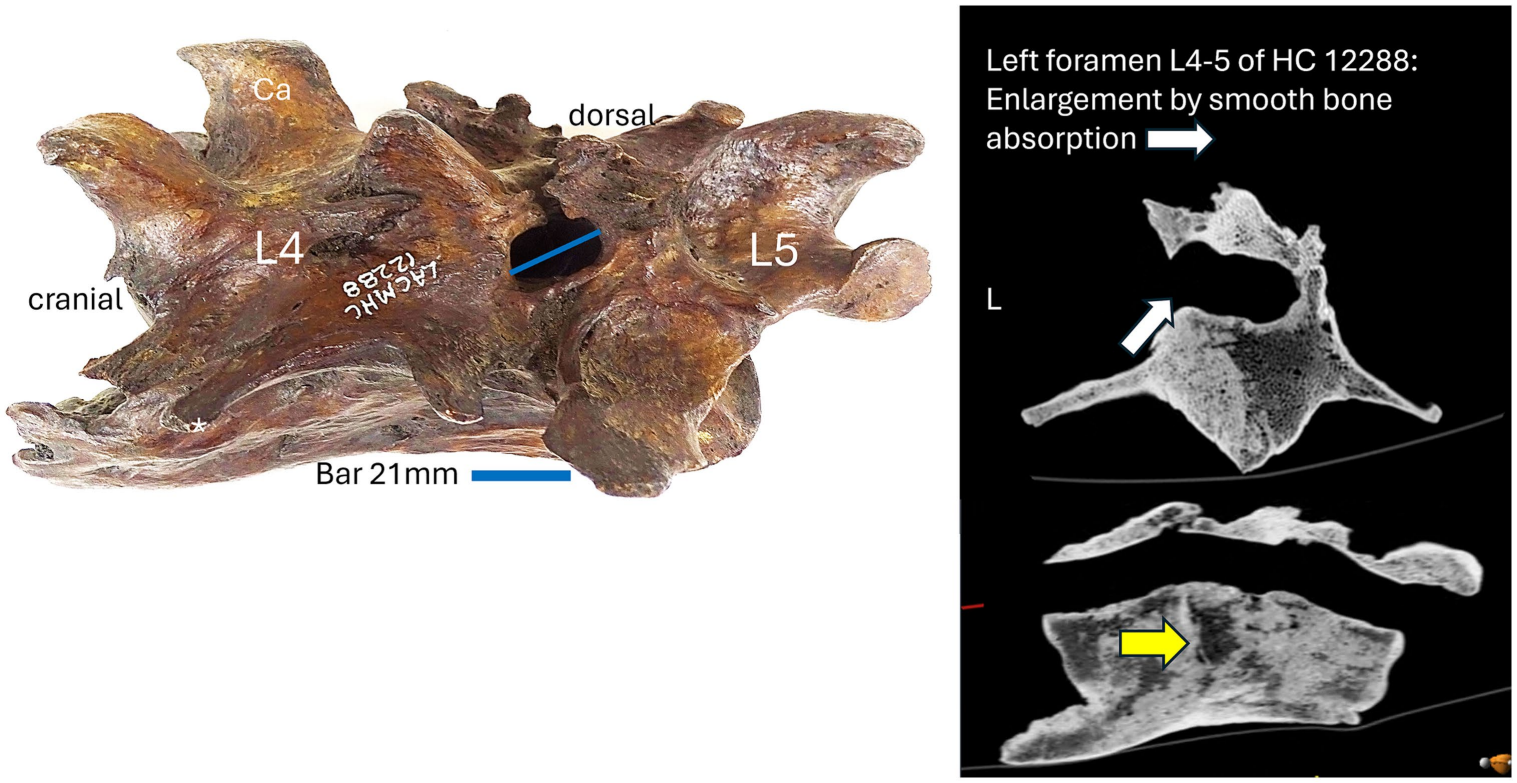
Photographs showing a lateral view and transverse and sagittal CT images of the HC 12288 lumbar segment presenting with L4–5 left-sided foraminal widening (white arrow) and fusion to form a block vertebra (yellow arrow).

**Figure 3:**
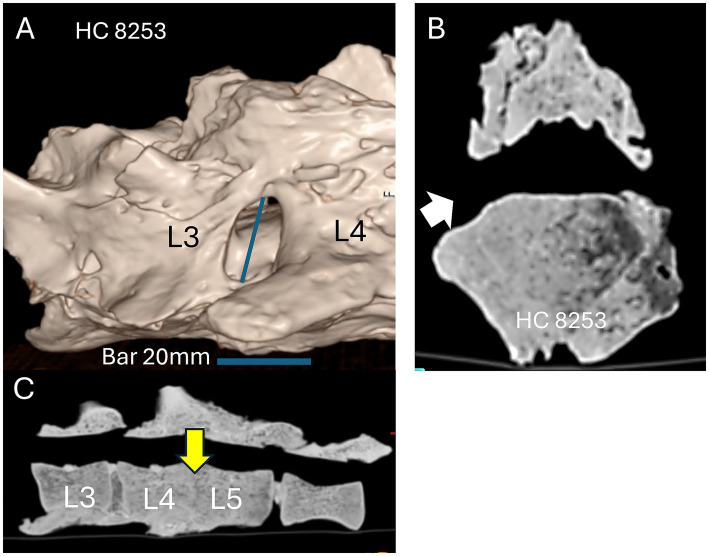
Volume-rendered three-dimensional (3D) reconstruction **(A)** and transverse **(B)** and sagittal **(C)** CT images of HC 8253 with left-sided foraminal widening at L3–L4 (white arrow) and fusion of L4–L5 to form a block vertebra (yellow arrow).

**Figure 4:**
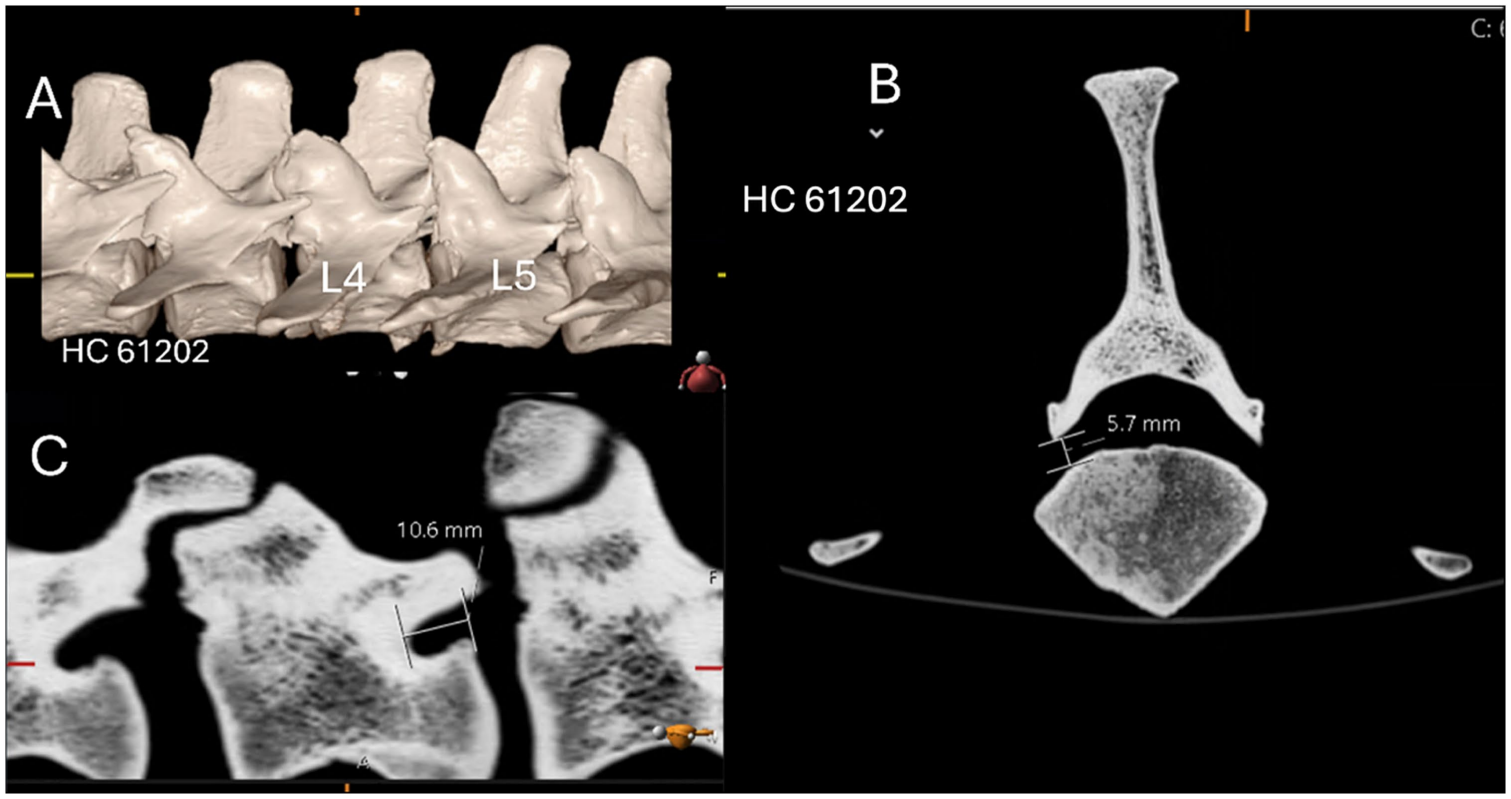
3D reconstruction **(A)** and transverse **(B)** and sagittal **(C)** CT image of HC 61202 with normal anatomy and a foraminal size L4–L5 of 10 mm x 6 mm.

Specimens HC 12288 and HC 8253 exhibit several severe pathologies, including congenital vertebral fusion forming block vertebrae and abnormal bone proliferations (10). Additionally, enlarged intervertebral foramina are present with smoothly marginated contours, likely caused by bone atrophy. These foraminal widenings, measuring 20 mm x 10 mm, can be interpreted as a dumbbell sign, which is a typical radiographic feature of an SNT (Figures 2, 3). The normal shape and size of a *S. fatalis* lumbar foramen are shown in Figure 4.

## Discussion

In *S. fatalis,* numerous transitional vertebrae have been documented, and there is also a high incidence of other congenital malformations such as bifid arches and block vertebrae (10). In this study, we describe evidence of another spinal lesion, compatible with and suggestive of a spinal nerve tumor, which is generally rare in occurrence. As is the case with all fossils from the LBTPM, no soft tissues are preserved. The bone pathologies can only be compared to those observed in extant species for which diagnoses are based on clinical signs, complete diagnostic imaging, and soft tissue examination, such as biopsy and histology of enlarged nerves. According to Kivrak et al. (23), foraminal widening and smooth bone absorption or remodeling along the anatomically normal course of a spinal nerve are rare diagnostic findings and are caused by SNTs in nearly all patients. It is reasonable to hypothesize, therefore, that this was also the case for the described *S. fatalis* specimens.

SNTs are very uncommon in humans and are estimated to occur in 0.22–0.38 per 100,000 persons (26–28). SNTs growing inward from or outward through the intervertebral foramen can widen the foramen and form so-called dumbbell-shaped tumors (19, 23). The vast majority of cases of foraminal widening in humans are caused by SNTs; other causative pathologies, such as bone tumors, tuberculous spondylitis, or infraforaminal synovial cysts, have been described but are extremely rare. Another potential cause of the described enlargement of an intervertebral foramen could be a pathology other than neoplasia, such as severe and chronic neural or perineural thickening with an underlying inflammatory pathophysiology (23, 29).

Multiple other mammalian species have been reported to be affected by SNTs. Goats, horses, and cattle suffer from peripheral SNTs such as Schwannomas, but no foraminal widening has been described (30–32). Typically, the affected peripheral nerve is evenly thickened. More information is available about SNT in dogs. The anatomical distribution is comparable to that in humans, with the brachial plexus being the most commonly affected site (18, 33, 34). Approximately 30–50% of the SNTs described in dogs extend through the foramen and can cause widening of the intervertebral foramen, which can be considered a significant radiographic sign (19, 20, 22). The widening is typically caused by slow nerve dimension growth, with no or limited bone infiltration, resembling pressure lysis or resorption without evident bone disruption.

The extant domestic cat, *Felis catus*, is affected by tumors of the nervous system, but SNTs are very rare (1, 21). The published cases of SNT in cats were mostly peripheral; only 3% of the reported cases in the literature originated in the spinal canal (25).

In the La Brea Tar Pits Museum database, there are 849 sacra registered. With three lumbar specimens showing foraminal widening among these individuals, the prevalence of this pathological finding is 353 per 100,000 *S. fatalis,* high compared to the 0.22–0.38 per 100,000 in humans (26–28). The reason for the high incidence of foraminal widening in the lumbar spine of *Smilodon fatalis* is unclear.

One in 2,500 humans is affected by a mutation of the tumor suppressor gene *NF1*. The mutation is inherited familiarly in half of the carriers; the others suffer from a spontaneous heterozygous loss-of-function mutation (35–38). Affected persons can develop malignant SNTs (23, 39). In Danish Holstein cattle, lineages derived from a few bulls have been described with significantly more peripheral SNT (30). Some genetic changes have been found in dogs with SNT, but as in Holstein cattle, no clear *NF1* mutation can be identified (30).

The high prevalence of foraminal widening in the *Smilodon fatalis* specimens from La Brea could be caused by the fact that these SNTs, at least in dogs, are often painful, cause lameness and muscle atrophy, and could impair successful hunting (40). In humans, approximately 75% of all patients with SNT (benign or malignant) have pain in some context (39). The large predators in the Pleistocene La Brea tar pits were attracted by the trapped herbivorous prey. This attraction must have been even stronger for diseased *Smilodon* individuals, as their hunting abilities were impaired, thereby increasing the necessity to venture into the tar pit.

Another possible cause could be a mutation like the one reported in human medicine. There is evidence that due to dramatically declining population numbers, there was an increasing occurrence of inbreeding in mammals at the end of the Pleistocene, with different health consequences. The cervical transitional vertebrae in the giant deer, woolly rhinoceros, and mammoth are an example (41–43). DNA analysis of mammoths found in the permafrost revealed a high degree of inbreeding before they became extinct, potentially causing serious diseases such as diabetes mellitus and sensory deficits (44). The Saber-toothed cats from La Brea suffered from osteochondrosis, resulting in defects in the subchondral bone, an inherited disease known in extant mammals and humans (17). Lumbosacral transitional malformations are associated with inbreeding in the extant gray wolves, and they were also found in *S. fatalis* (2, 3, 10). A genetic predisposition associated with population decline or inbreeding is a plausible but currently untested hypothesis for the observed high prevalence of possible SNT in the lumbar spine of the *Smilodon fatalis.* Without DNA analysis, the exact degree of inbreeding of the animals found in the tar pits of La Brea is unknown. Inbreeding, infectious diseases, or toxic environmental agents have been discussed as causes for the malformations found in the La Brea *S. fatalis* (10). Congenital malformations and tumors often have a complex molecular basis that cannot be explored in fossils lacking DNA for analysis. The described changes found in the vertebrae of the *S. fatalis* are a strong indicator that SNTs may have been present and may have been an important disease process at the end of their existence.

## Data Availability

The datasets presented in this study can be found in online repositories. The names of the repository/repositories and accession number(s) can be found at: https://doi.org/10.5281/zenodo.17688403.

## Footnotes

1
www.horosproject.org
